# Molecular Evolutionary Landscape of the Immune Microenvironment of Head and Neck Cancer

**DOI:** 10.3390/biom13071120

**Published:** 2023-07-14

**Authors:** Baoyi Shao, Zheng Ye, Bo Sun, Zhongdang Xiao

**Affiliations:** State Key Laboratory of Bioelectronics, School of Biological Science and Medical Engineering, Southeast University, Nanjing 210096, China

**Keywords:** immune subtype, HNSC, tumor immune microenvironment, immune landscape, immunotherapy

## Abstract

Head and neck cancer is a highly heterogeneous malignant tumor. Numerous studies have shown that the immune microenvironment of head and neck cancer has a significant impact on its occurrence and development, as well as its prognosis. However, there have been fewer studies related to the accurate immunophenotyping of head and neck cancer. In this study, we used gene expression profile information and clinical information from the TCGA-HNSC cohort (502 samples) and the GSE655858 cohort (270 samples) to identify and independently validate three immune subtypes (Cluster1–Cluster3) with different immune-related molecular profiles and clinical outcomes. Cluster2, which is mainly dominated by B-lymphocyte infiltration, was found to have the best prognosis. In addition, a support vector machine (SVM)-based classifier was constructed, which could accurately classify HNSC based on 19 genes. Furthermore, the results of the prognostic analysis showed activation of antibody-secreting B-lymphocyte function, which showed a good prognostic effect in all three immune subtypes of HNSC. Finally, the immune evolutionary landscape of HNSC was constructed in an attempt to explain the evolutionary pattern of the immune subtypes of HNSC. In summary, we provide a conceptual framework for understanding the tumor immune microenvironment in HNSC and demonstrate the importance of immune infiltration of B lymphocytes in HNSC. Further research is needed to assess the importance of these immunophenotypes in combination drug therapy and to provide a basis for screening appropriate patients for immunotherapy.

## 1. Introduction

Head and neck cancer is a malignant tumor with a high global incidence, ranking as the sixth most common cancer worldwide [1,2,3]. The occurrence of head and neck cancer is highly correlated with smoking, alcoholism and HPV infection [4,5,6]. Over 90% of head and neck cancers are squamous cell carcinomas [7]. Despite significant advancements in the treatment of head and neck cancer in recent years, the prognosis for survival remains low [8]. In the early stages of head and neck cancer, surgery and radiotherapy serve as the primary treatments. However, by the time head and neck cancer is diagnosed, over 50% of patients are at an advanced clinical stage and have missed the optimal window for treatment [9]. The prognosis for patients with refractory and recurrent head and neck cancer is even worse [10].

Immune checkpoint inhibitors (ICIs), such as those targeting programmed death-ligand 1 (PD-L1), programmed death 1 (PD1), and CTL-associated protein 4 (CTLA4), have shown greater efficacy than traditional therapies [11,12]. PD1 anti-therapy has demonstrated promising results for recurrent/metastatic head and neck squamous cell carcinoma (HNSC) [13]. A major limitation of ICI therapy is the low response rate of patients. The tumor microenvironment (TME) is one of the factors that can affect the effectiveness of an ICI, and only a few biomarkers can accurately predict the patient’s prognosis [14,15]. HNSC patients can benefit from individualizing immunotherapy with the assistance of potential prognostic markers. In recent research on the immune microenvironment in HNSC, Mandal’s work has suggested that molecular characterization of HNSC tumors should be an integral and potentially novel approach for investigating agents targeting modulators of Tregs and NKs [16].

**Figure 1:**
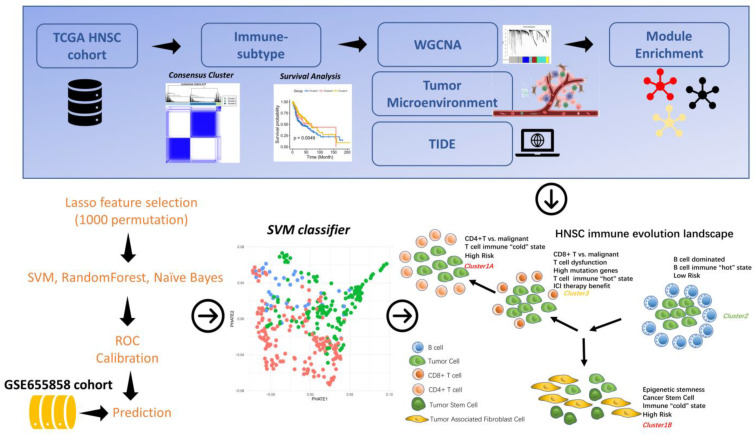
Flow chart of this study.

The objective of this study was to examine the immune subtypes of head and neck cancer and to construct molecular evolutionary profiles of these immune subtypes to explore the tumor microenvironmental features and validate their reproducibility in independent cohorts. By performing consistent clustering using immune-related genes, we identified three immune subtypes and five immune-related functional modules. Each immune subtype corresponds to a different clinical, molecular and cellular characteristic. We found that each of the three immune subtypes was associated with different gene expression patterns, molecular and cellular features, and clinical outcomes. Our findings have unveiled the distribution characteristics of the complex tumor microenvironment in HNSC patients and have provided insight into the molecular evolution of tumors. The workflow of this study is shown in Figure 1.

## 2. Methods

### 2.1. Data Extraction

Gene expression profile matrices and clinical information were obtained from The Cancer Genome Atlas (TCGA [17], https://www.cancer.gov/tcga, accessed on 22 September 2022) via the GDC API [18]. GSE65858 was downloaded from the GEO database [19]. The single cell sequencing dataset was retrieved from the TISCH database (http://tisch.comp-genomics.org/gallery/?cancer=HNSC, 22 September 2022). Clinical information about the sample is available in Supplementary File S1. A total of 2483 immune-related genes were obtained from ImmunePort [20] (https://www.immport.org/resources, accessed on 22 September 2022). These immune-related genes include antigen processing and presentation, antimicrobials, BCR signaling pathway, chemokine receptors, chemokines, cytokine receptors, cytokines, interferons, interferon receptors, interleukins, interleukin receptors, natural killer cell, TCR signaling pathway, TGF-β family members, TGF-β family members receptors, TNF family members, TNF family members receptors. 1722 of these genes overlap with the expression profile matrix.

### 2.2. Data Preprocessing

Data from samples lacking clinical information and normal samples were excluded first. The FPKM matrices of HNSC samples were converted to TPM matrices and log2 (TPM+1) transformations were performed. The expression profile matrices of 1722 genes overlapping with 2483 immune-related genes were selected for subsequent analysis.

### 2.3. Gepia Analysis

Differential gene expression and patient survival data were obtained using Gene Expression Profiling Interactive Analysis [21] (GEPIA, version 2, http://gepia2.cancer-pku.cn, accessed on 3 October 2022). The LIMMA [22] was used to identify differentially expressed genes with |log2FC| > 1 and q values < 0.01. OS and disease-free survival (DFS) were evaluated using the Kaplan–Meier method with a 50% (Median) cutoff and compared by the logrank test. The cox proportional hazards regression model was used to get the hazard ratio. *p* values < 0.05 were considered statistically significant.

### 2.4. cBioPortal Analysis

The cBio cancer Genomics Portal [23] (cBioPortal, http://www.cbioportal.org, accessed on 3 October 2022) was used to integrate the TCGA dataset and compare genetic alterations in HNSC. *p*-value < 0.05 was considered statistically different.

### 2.5. TIMER Analysis

The Tumor Immune Estimation Resource [24] (TIMER, https://cistrome.shinyapps.io/timer/, accessed on 6 October 2022) was used to analyse and visualize the correlation between tumor immune infiltrating cells and genes (Spearman). *p* < 0.05 was considered statistically significant. Tumor immune infiltration, tumor mutational load and microsatellite instability data were obtained from The Immune Landscape of Cancer [25] (https://gdc.cancer.gov/about-data/publications/panimmune, accessed on 6 October 2022) database. In addition, tumor stemness was obtained from the PanCanStemness [26] (https://gdc.cancer.gov/about-data/publications/PanCanStemness-2018, accessed on 6 October 2022) database.

### 2.6. The Immune Subtypes

The 1722 immune-related genes were clustered according to their expression profiles and a consistency matrix was constructed to identify the corresponding immune subtypes and co-expressed gene modules. The divisive hierarchical clustering algorithm [27] was applied to calculate the distance between samples and a 1000 bootstrap was performed, using 80% of the patients in the cohort for each run. The number of clusters was set to a range of 2 to 6 and the optimal number of clusters was selected by evaluating the consistency matrix and the consistency cumulative distribution function.

### 2.7. Construction of Immune Landscape

Monocle [28] was used to perform non-linear dimensionality reduction on the expression profile matrix. The DDRTree algorithm [29] was used to construct the pseudo-temporal progression and bifurcation structure of the immune-related gene expression profile of HNSC patients. The pseudo-time progress map was constructed by selecting high-variability genes. Expression profile variation analysis was performed by branched expression analysis modeling (BEAM) on branches of nodes.

### 2.8. Prognostic Evaluation of Immune Subtypes

The prognosis of immune subtypes was evaluated using the log-rank test and univariate cox regression model, with age as a covariate, and OS and DFS as the focus. xCell [30] was used to evaluate 33 immune-related cells in the sample. The Weighted Gene Co-Expression Network Analysis algorithm (WGCNA) [31] was used to cluster genes. clusterProfiler [32] was used to perform functional enrichment analysis on the hub genes of each module.

### 2.9. Construction of Machine Learning Classifier

Orange3 [33] (version: 3.28) was used to construct a machine learning classifier. The 1090 genes with a standard deviation greater than 1 were first selected, and then the top 100 genes were selected using the Rank widget of Orange3 (using Info.gain, Gain ratio, Gini, ANOVA, and chi-square). Finally, using Logistic Regression (Ridge, C = 1) with 1000 permutations, the 15 genes that contributed most to the classification task were found as the set of feature genes. The sample was randomly divided into 70% training set and 30% test set. Support vector machines, random forests, and Naïve Bayes algorithms were used in the training set. A 5-fold cross validation was used to evaluate the performance of the model.

## 3. Results

### 3.1. Identification of Potential HNSC Immune Subtypes

**Figure 2:**
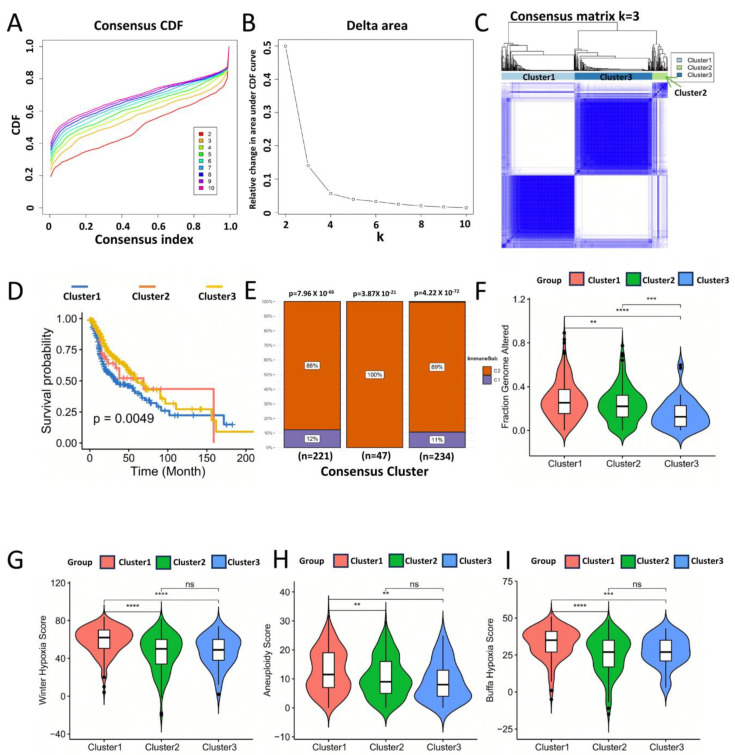
Identification of potential immune subtypes of HNSC. (**A**) Cumulative distribution function curve and (**B**) delta area of immune-related genes in TCGA cohort. (**C**) Sample clustering heatmap. (**D**) Kaplan–Meier curves showing OS of HNSC immune subtypes in TCGA cohort (Logrank Test). (**E**) Comparison of immune subtypes obtained from HNSC with those from ImmuneSubtypeClassifier. The HNSC was mainly C1 and C2 subtypes, with the highest percentage of C2 subtypes. In addition, Cluster2 was all C2 subtypes. (**F**–**I**) Fraction Genome Altered percent, Winter Hypoxia Score, Aneuploidy Score and Buffa Hypoxia Score have significant differences in different HNSC immune subtypes. The mean values of these indicators in Cluster1 are highest (Student’s *t*-test). ** *p* < 0.01, *** *p* < 0.001, **** *p* < 0.0001 and ns: not significant.

The immune subtype of HNSC can reflect the immune status in the tumor microenvironment, thereby helping to identify suitable patients for immunotherapy. We analysed the expression profiles of 1722 immune-related genes in 502 HNSC samples from the TCGA database for the construction of consistency clusters. Based on their consensus CDF and delta area, we chose k = 3 (Figure 2A,B). Eventually, we obtained three immune subtypes named Cluster1–Cluster3 (Figure 2C). The Kaplan–Meier survival analysis showed that Cluster2 had the best prognosis, Cluster1 the worst, and Cluster3 was in between (hazard ratio: Cluster 2 > Cluster 3 > Cluster 1). There is a significant difference in survival between the three immune subtypes (*p* = 0.01) (Figure 1D). The immune subtypes of HNSC were evaluated by ImmuneSubtypeClassifier. The results showed that type C2 (IFN-γ dominant) had the highest percentage of the three subtypes (88% in Cluster1, 100% in Cluster2, and 11% in Cluster1). In addition, 12% of Cluster1 belonged to C1 type (wound healing type) and about 11% of Cluster3 belonged to C1 type (Figure 1E). Differences in clinical characteristics between the three immune subtypes were analysed using the cBioPortal database. The results showed that the Fraction Genome Altered, Winter Hypoxia Score, Aneuploidy Score and Buffa Hypoxia Score of Cluster1 were significantly higher than the other two immune subtypes (Figure 2G–I). These results illustrate the significant heterogeneity of the HNSC immune subtypes we obtained by consistent clustering.

### 3.2. Head and Neck Cancer Immune Subtype of Gene Mutation Characteristics

**Figure 3:**
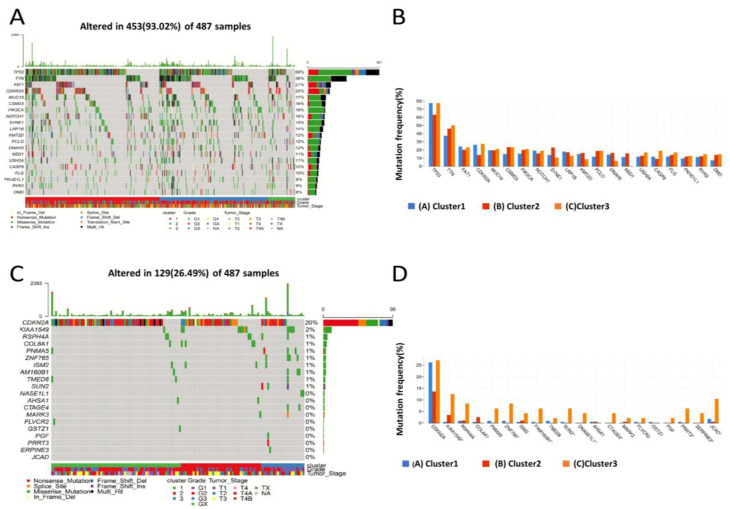
Top 20 significant alteration event frequency genes in different clusters. (**A**) Frequency distribution of alteration events for the genes with the highest mutation frequencies in different clusters. (**B**) Mutation landscape of the genes with the highest mutation frequencies. (**C**) Distribution of mutations in genes with significantly different mutation frequencies among different clusters. (**D**) Mutation landscape of genes with significantly different mutation frequencies. Wilcoxon test, * *p* < 0.05.

To investigate the relationship between immune subtypes and tumor genomic mutations, we selected the vanscan2-processed TCGA mutation dataset and calculated the TMB and mutation count for each patient. We found no apparent differences in TMB and mutation count for these subgroups (data not shown). However, TP53 and TTN remained the two genes with the highest mutation rates among the three immune subtypes (Figure 3A,B). In addition, the number of genes mutated in Cluster3 was much higher than in the other subtypes (Figure 3C,D), with a significant difference in the high mutation rate of CDKN2A between the three subtypes (*p* < 0.01). These findings suggest that there are significant differences in gene mutation profiles between the three immune subtypes.

### 3.3. Immune Modulators and Tumor Microenvironment Characteristics of Immune Subtypes of Head and Neck Cancer

**Figure 4:**
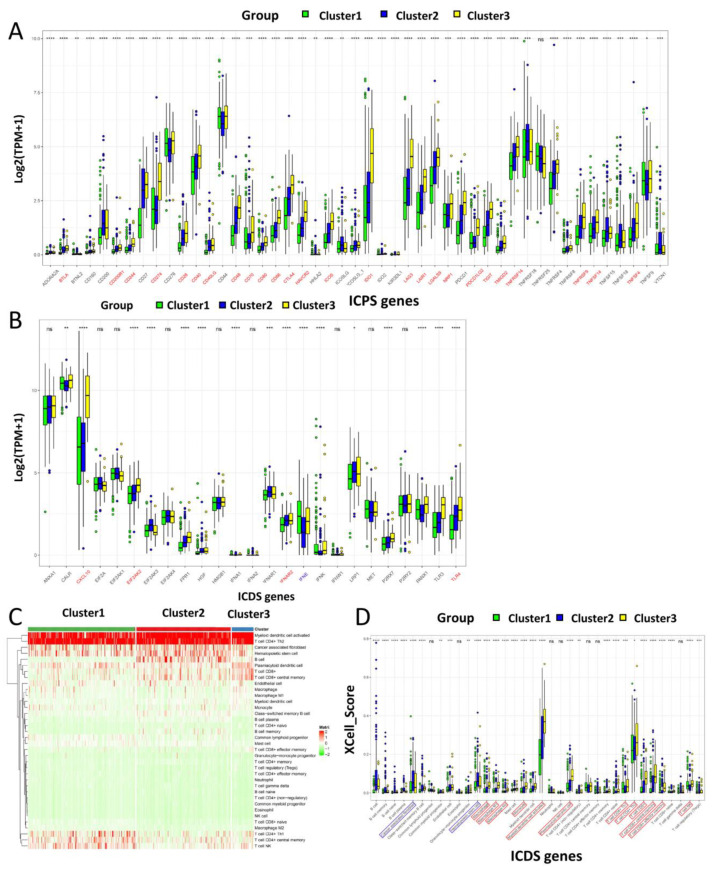
Association between immune subtypes and ICP and ICD modulators and cellular characteristics of immune subtypes. (**A**) Differential expression of ICP module genes in HNSC immune subtypes. (**B**) Differential expression of ICD module genes in HNSC immune subtypes. xCell was used to calculate the scores of 33 common immune cells. (**C**) The distribution of immune cell infiltration scores in the three clusters. (**D**) Comparison of the differences in immune cell infiltration scores between the three clusters. Kruskal–Wallis test, * *p* < 0.01, ** *p* < 0.001, *** *p* < 0.0001 and **** *p* < 0.00001.

Immune checkpoints and immunogenic cell death modulators are of great significance in cancer immunity. Next, we compared the expression levels of genes in these modulators in different subtypes. In this cohort, 47 genes associated with ICPs (immune checkpoints) were detected (Figure 4A). Genes, which were marked in red, including BTLA, CD200R1, CD244, CD274, CD28, CD40, CD40LG, CD48, CD70, CD80, CD86, CTLA4, HAVCR2, ICOS, IDO1, LAG3, LAIR1, LGALS9, NRP1, PDCD1LG2, TIGIT, TMIGD2, TNFRSF14, TNFRSF9, TNFSF14 and TNFSF4 expression increased significantly in Cluster3 immune subtypes, but significantly decreased in Cluster1. The 25 genes related to ICD (immunogenic cell death) also showed significant heterogeneity in the three immune subtypes (Figure 4B). The four genes CXCL10, EIF2AK2, IFNAR2 and TLR4 (marked in red) were significantly increased in the Cluster3 immune subtype, but dramatically decreased in the Cluster1 immune subtype. Additionally, we discovered that the expression level of IFNE (marked in blue) in Cluster2 was significantly reduced, while it was significantly increased in Cluster1. Thus, immunophenotyping can reflect the expression levels of ICP and ICD modulators in HNSC patients and can be used to assess the activation status of the tumor immune microenvironment in these patients. Next, we used xCell to assess the status of 33 immune cell types in HNSC tumor tissue in these three immune subtypes. The three immune subtypes showed significant differences from each other. Immune activation in Cluster1 was mainly dependent on CD4+ Th1, CD4+ central memory T cells and NKT cells. The immune activation of Cluster2 was mainly dependent on B cells, class-switched memory B cells and plasma B cells. The immune activation of Cluster3 was mainly related to CD8+ T cells and CD8+ central memory T cells (Figure 4C). According to Figure 4D, it can be seen that activated Myeloid dendritic cells, CD8+ T cells, CD8+ central memory T cells, Macrophage, Macrophage M1, Macrophage M2, Monocyte, Plasmacytoid dendritic cells and other cells (red border) were found to be highly immune infiltrated in the Cluster3 immune subtype. B cells, cancer associated fibroblasts and hematopoietic stem cells (bule border) have a higher degree of immune infiltration in Cluster2. In Cluster1, the immune infiltration was relatively low. This could explain the lower prognostic survival of Cluster1 and the higher prognostic survival of Cluster3. The Cluster1 immune subtype is in an immune “cold” state.

### 3.4. Immune Landscape of HNSC

**Figure 5:**
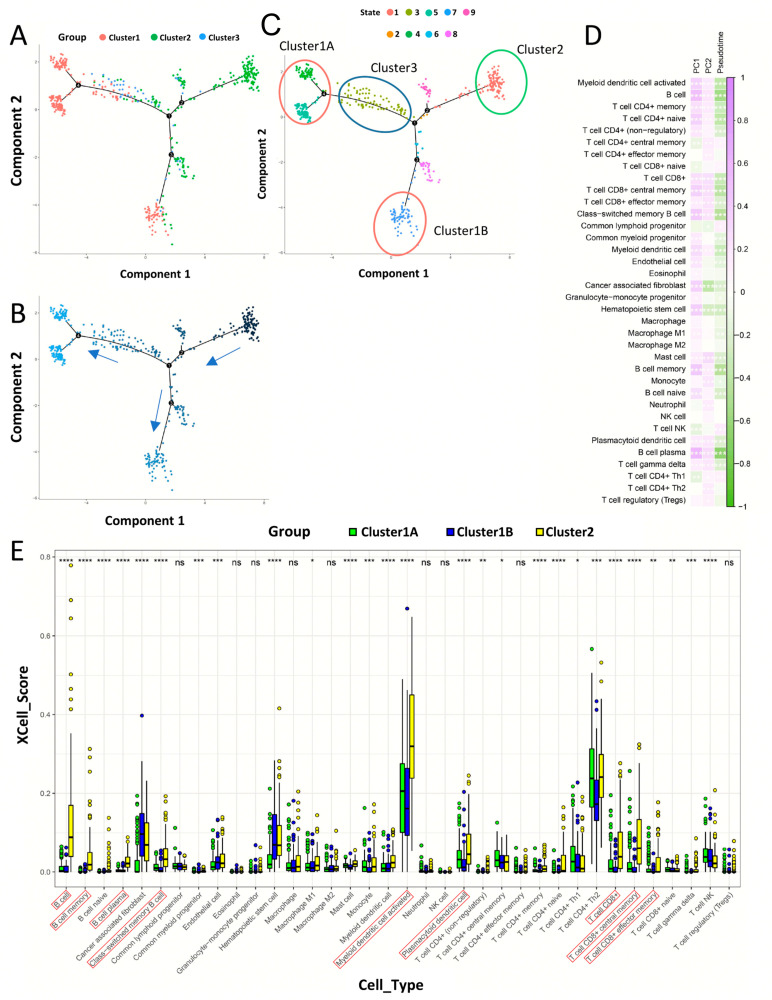
Immune landscape of HNSC. (**A**) Immune landscape of HNSC. Each dot represents a patient, and the color represents the immune subtype to which it belongs. (**B**) Immune subtype differentiation trajectory diagram. (**C**) Nine states of patients obtained using monocle’s DDRTree algorithm. Cluster1 contains three states, state4, state5 and state7; Cluster2 mainly has one state, state1; Cluster3 is mainly distributed in state3. State4 and state5 are considered to be an immune subtype of Cluster1 and named Cluster1A. State7 is considered to be another immune subtype of Cluster1 and is named Cluster1B. The immune subtype of HNSC differentiated from the beginning of state1 to the change process of the terminal expression profile of state4, state5 and state7. (**D**) Correlation between Comp1, Comp2, Pseudotime and 36 immune cell scores (xCell). (**E**) Comparison of the mean values of the xCELL scores of 33 immune cells in the 3 immune subtypes. Correlations were performed using the Pearson correlation coefficient, and group comparisons were performed using the Kruskal–Wallis test. * *p* < 0.01, ** *p* < 0.001, *** *p* < 0.0001 and **** *p* < 0.00001.

The immune gene expression profile of 502 HNSC patients was used to construct the HNSC immune landscape (Figure 5A). According to the different immune statuses of HNSC patients, the DDRTree algorithm divides HNSC patients into 9 states. The Cluster1 immune subtype mainly includes state4, state5 and state7; the Cluster2 immune subtype mainly includes state1; and the Cluster3 immune subtype is mainly distributed in state3. Pseudotime is used to simulate the evolution of tumor immune environment in HNSC patients (Figure 5B).

**Figure 6:**
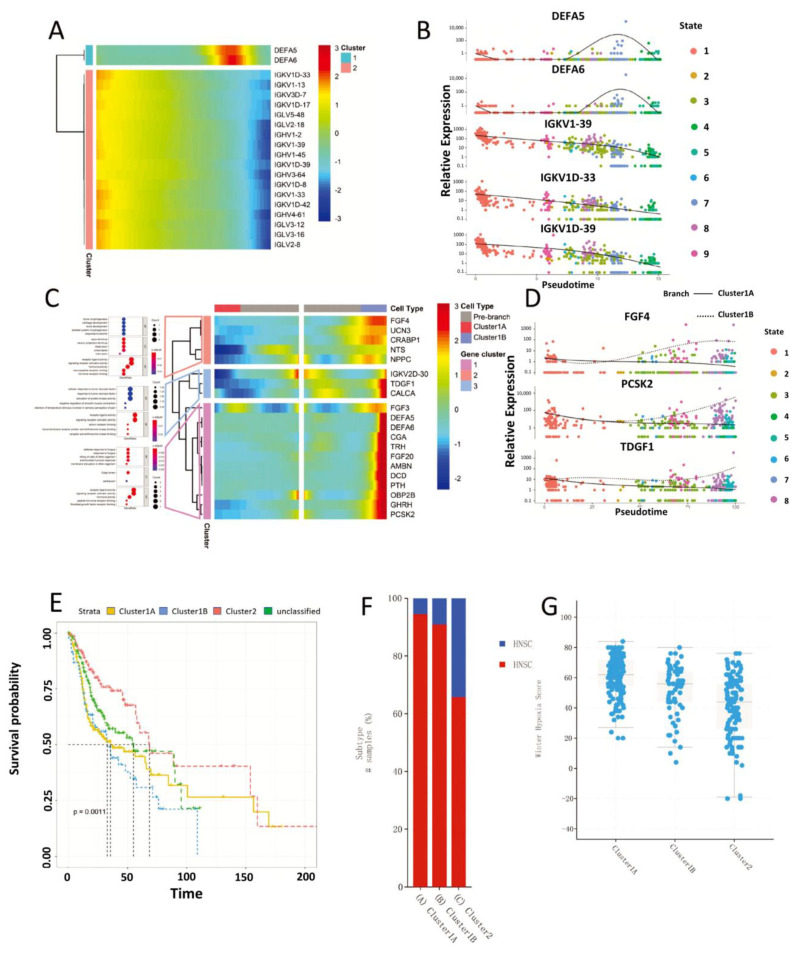
Distribution map of characteristic gene variation of immune subtypes. (**A**) Heat map of the top 20 genes (with the largest changes in expression) of HNSC patients during the transition from Cluster1 to Cluster2 along the Pseudotime trajectory. The abscissa axis represents Pseudotime. (**B**) The distribution of the top5 highly variable genes in 9 states along Pseudotime in (**A**). (**C**) The top20 gene heat map during the differentiation of Cluster2 into Cluster1A and Cluster1B along Pseudotime, and the functional enrichment results (KEGG) of each cluster. (**D**) From the three gene clusters in (**B**), the selected genes with the most significant variation and their plotted distribution in the 9 states along Pseudotime. The solid line represents the gene expression level change process of the Cluster1A immune subgroup, and the dotted line represents the gene expression change process of the Cluster1B immune subgroup. (**E**) The relationship between immune subtypes and clinical traits, and the relationship between immune subtypes and patient survival. The OS of the three groups is significantly different, and HR: Cluster2 < Cluster1A < Cluster1B. (**F**) The relationship between immune subtype and the patient’s HPV infection status. The proportion of patients with HPV infection belonging to the immune subtype of Cluster2 is higher. (**G**) The relationship between the immune subtype and the patient’s Winter Hypoxia Score. The Winter Hypoxia Score of Cluster2 patients was lower. There are significant differences between the three groups of immune subtypes (q value < 2.17× 10^−13^).

Further immuno-subtyping groupings were made based on samples in the top position in the immunotyping landscape. State1 was named DDRT_Cluster2, State4 and State5 were named Cluster1A, and State7 was named Cluster1B (Figure 5C). Both Component1 and Component2 showed a significant correlation with immune infiltration of multiple immune cells, while Pseudotime had a negative correlation. T cell CD4+ effector memory showed a negative correlation with Component1 and a positive correlation with Component2. Cancer-associated fibroblast and hematopoietic stem cells were positively correlated with Component1 and negatively correlated with Component2 (Figure 5D). Combining the previous findings, we found that Cluster1A is mainly characterized by immune infiltration of CD4+ central memory T cells, NK T cells and Th1 CD4+ T cells (*p* < 0.01); Cluster1B is mainly characterized by immune infiltration of cancer associated fibroblasts and hematopoietic stem cells in the tumor microenvironment; Cluster3 was mainly characterized by immune infiltration of activated myeloid dendritic cells, B cells, CD8+ T cells, CD8+ central memory T cells, CD8+ effector memory T cells, class-switched B cells, myeloid dendritic cells and plasmacytoid dendritic cells (Figure 5E). Furthermore, in view of the immune evolutionary process simulated by the immune subtypes of HNSC, the immune gene expression profile of HNSC patients changed significantly with the progression of Pseudotime. Among the top 20 most significantly mutated genes, we identified a trend towards obviously lower expression of immunoglobulin-related genes such as IGKV1-39, IGKV1D-33 and IGKV1D-39; while neutrophil-related genes such as DEFA5 and DEFA6 were highly expressed in Cluster1B and lowly expressed in Cluster1A (Figure 6A,B). To further elucidate the differences between Cluster1A and Cluster1B during the molecular evolution of immune subtypes, we analysed branching node 1. The results revealed that the immune genes of Cluster1B are differentially elevated and those of Cluster1A are decreased during the differentiation of HNSC from the low-risk Cluster2 to both Cluster1A and Cluster1B states (Figure 6C,D). This reflects the difference in immune status exhibited by Cluster1A and Cluster1B. The cBioPortal database was used to compare the differences in clinical characteristics between Cluster1A, Cluster1B and Cluster2. It turned out that Cluster2 had a significantly higher survival rate than Cluster1A and Cluster1B. The three subgroups had significantly different disease-specific survival, HPV status, and Winter Hypoxia Score logrank test *p*-value < 0.001(Figure 6E–G). In addition, we obtained the tumor stem cell index scores of these HNSC samples through mRNA expression profile data. By comparing the differences between Cluster1A, Cluster1B and Cluster2 (Wilcoxon test), we found that the EREG-mRNAsi of the Cluster2B sample was significantly higher than that of the other two groups, while the mRNAsi was the opposite (Figure S1). The above results suggest that the evolution of Cluster2 to Cluster1B involves not only a ‘cooling’ of the immune state but also an increase in tumor stemness caused by epigenetic modifications. It may be a sign of tumor transformation to a cancer stem cell state. This is indirectly evidenced by the increased immune infiltration of cancer-associated fibroblasts and hematopoietic stem cells in Cluster1B.

In summary, the immune landscape of HNSC can accurately identify the immune cellular components of HNSC patients and predict their prognostic survival status and, most importantly, characterize the molecular evolution of the cancer immune microenvironment in HNSC.

### 3.5. Construction of the HNSC Immune Subtype Classifier

**Figure 7:**
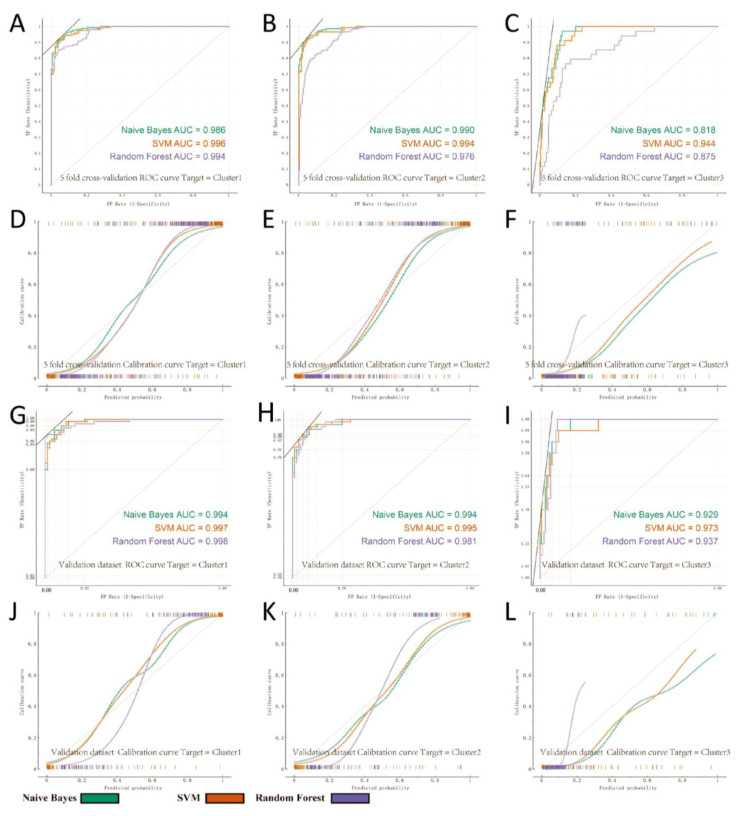
Comparison of machine learning models in the validation set. (**A**,**D**,**G**,**J**), (**B**,**E**,**H**,**K**) and (**C**,**F**,**I**,**L**) are the ROC curves and calibration curves of Cluster1, Cluster2 and Cluster3 classifiers, respectively. We compared three machine learning algorithms and evaluated the classification performance of the models using several metrics and ultimately found that SVM performed the best (Supplementary File S2).

The expression profile matrix of HNSC samples was used to accurately classify the immune subtypes of HNSC patients, enabling targeted ICI therapy. We built a stable classifier using the three immune subtypes (Cluster1, Cluster2 and Cluster3) obtained by consistent clustering. Firstly, we normalized the TCGA-HNSC dataset using voom (R package LIMMA) and then used the removeBatchEffect function to merge the GSE65858 dataset and remove the batch effect. Next, we filtered the TCGA-HNSC dataset for genes with a standard deviation greater than 1.0 (1090, see Supplementary File S1). These genes were ranked using the Orange3 rank widget, and the top 100 genes were chosen for subsequent feature selection (see Supplementary File S1). To select the most effective features for classification, we performed 1000 perturbations using Logistic Regression (L2 regularization) and ranked the top 20 genes based on mean feature weights. We selected the top 15 genes for the construction of the classification model (Figure S2A red markers). The TCGA-HNSC dataset was divided into a training set (70%) and a test set (30%), with GSE65858 as the prediction set. We compared the classifiers constructed using three machine learning models (Naïve Bayes, SVM and Random Forest) and finally found that the SVM classifier yielded the best results (Figure 7A–L). This demonstrates that the model constructed using the SVM classifier can accurately classify the immune subtypes of HNSC. 20 feature genes were filtered by SVMRFE algorithm to build classifiers for cluster1A, cluster1B, cluster2 and unclassified (Table S1). Tables S2 and S3 showed the performance of three machine learning models (SVM, Random Forest and Naïve Bayes) in training and test set respectively. Moreover, we observed that CD79A and IGJ had the highest weighting among the 15 characterized genes screened, since CD79A and IGJ are both molecular markers for B-cells and plasma cells. In addition, we verified the difference in xCell scores between the three clusters in the GSE65858 dataset (Figure S3A,B). The results were consistent with the previous ones, with Cluster3 having the most pronounced immune activation profile and Cluster1 the worst. This indicates that immune infiltration of cells associated with antibody secretion is important for the typing of immune subtypes of HNSC.

### 3.6. HNSC Immune Gene Co-Expression Module and the Single Cell Expression Profile Localization

**Figure 8:**
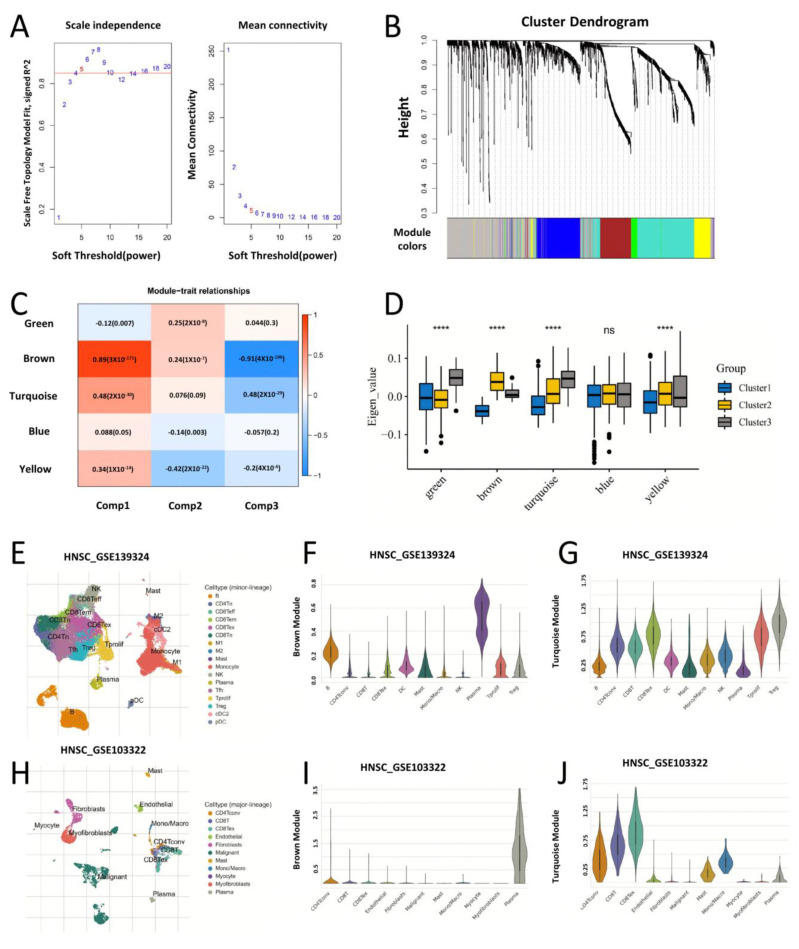
Identification of immune gene co-expression module of HNSC. (**A**) Scale-free fit index and mean connectivity for the soft-thresholding powers. (**B**) Dendrogram of immune genes clustered based on a dissimilarity measure (1-TOM). (**C**) Module–trait relationship matrix (Pearson correlation). (**D**) Differential distribution of module eigenvectors in each cluster (*t*-Test). **** *p* < 0.0001, ns: not significant. (**E**–**G**) Cellular localization of single-cell transcriptomes of co-expression modules. In the HNSC GSE139324 dataset, the hub genes (MM > 0.9) of the brown module are mainly enriched in plasma cells. The hub genes (MM > 0.9) of the turquoise module are mainly enriched in CD8+ T cells, especially the expanded CD8+ T. (**H**–**J**) In the HNSC GSE103322 dataset, the hub gene of the brown module is also enriched in the plasma cell. The co-expression network of the turquoise modules mainly occurs in T lymphocyte subsets. In the HNSC GSE103322 dataset, the hub gene of the turquoise module is mainly enriched in T lymphocytes. These results indicate that there is extensive activation of T lymphocytes in the tumor microenvironment.

Weighted Gene Co-Expression Network Analysis (WGCNA) was used to cluster the immune-related genes of HNSC samples, and the soft threshold of the scale-free network was set to 5 (Figure 8A). The expression profile matrix was transformed into a topological matrix, and average-linked hierarchical clustering was performed to construct a hierarchical clustering tree of genes. The tree was then pruned and modularly clustered using a hybrid dynamic shearing tree algorithm, with each module containing at least 30 genes. The Eigenvector was calculated for each module, and similar modules were merged with a height of 0.25 and a depth of 2 (Figure 8B). We finally obtained five co-expression modules, with genes in the grey module not clustered with genes in the other modules. Further, we analysed the Pearson correlation between the five modules and Component1, Component2 and Pseudotime (Figure 8C). The results show that the brown module and turquoise module have the strongest correlation with Component1 and Pseudotime (component1 cor. brown = 0.89, component1 cor. turquoise = 0.48, Pseudotime cor. brown = −0.91, Pseudotime cor. turquoise = −0.48). By comparing the module eigenvectors of the five modules among the three clusters, we found that Cluster1 was mainly characterized by co-repression of genes from the brown and turquoise modules; Cluster2 was mainly characterized by activation of the co-expression network of the brown module; and the main feature of Cluster3 was the activation of the green and turquoise modules (Figure 8D). The MM (Module Membership) of component1, component2 and Pseudotime in the brown module showed a significant correlation with the GS (Gene significance) of the genes (Component1: MM cor. GS = 0.98, Component2: MM cor. GS = 0.63, Pseudotime: MM cor. GS = 0.99) (Figure S4A,C,E). The MM and GS of component1 and Pseudotime in the turquoise module showed significant correlations (Component1: MM cor. GS = 0.69, Pseudotime: MM cor. GS = 0.76), while component2 did not (Figure S4B,D,F). In the brown module, we selected 45 genes with MM > 0.9 as the hub genes of the module. In the turquoise module, we selected 17 genes with MM > 0.9 as the hub genes for this module. The TISCH database was used for single cell transcriptome localization of these hub genes. The hub genes of the brown module are predominantly located in plasma cells, whereas the hub genes of the turquoise module are predominantly located in T lymphocytes. We validated two sets of HNSC single cell transcriptome sequencing data at GSE139324 and GSE103322, respectively, and obtained consistent results (Figure 8E–H,J). The result of GO function enrichment showed that the hub genes of the brown module were mainly related to complement activation, immunoglobulin-mediated immune response, B cell-mediated immunity and other signaling pathways; the hub genes of the turquoise module were mainly related to the T cell receptor signaling pathway, antigen receptor-mediated signaling pathway, and the immune response-activating cell surface receptor signaling pathway. These results are consistent with the previous conclusions (Figure S5A,B).

### 3.7. Prognostic Factors and Immunotherapy Benefits in Different Immune Subtypes

**Figure 9:**
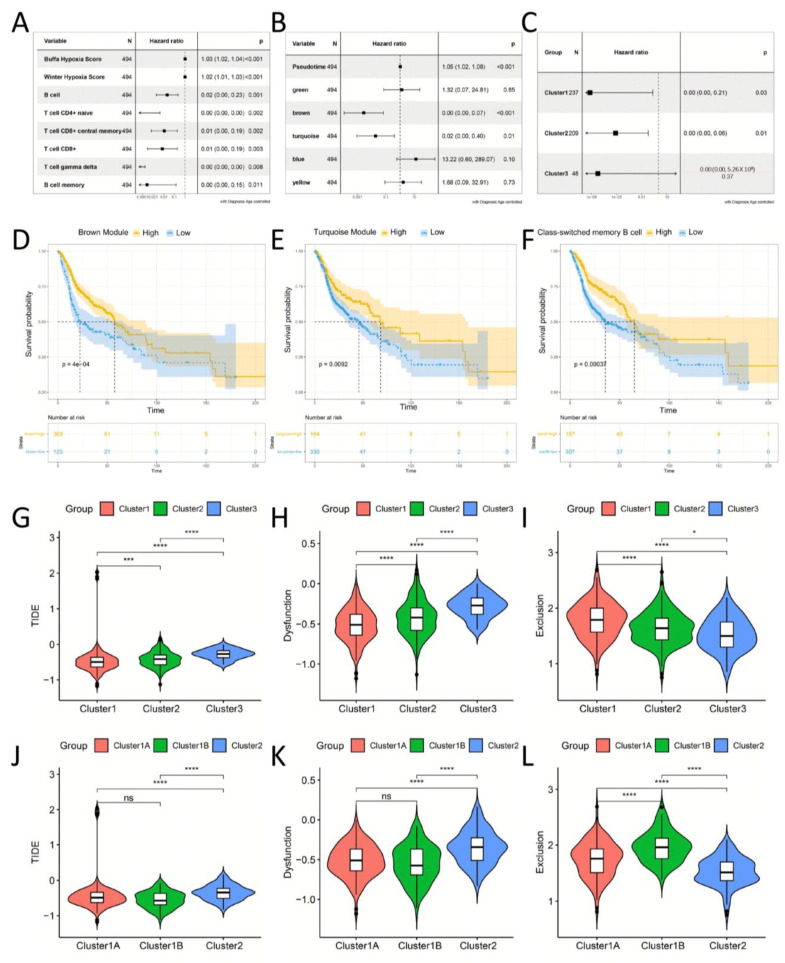
Identification of immune hub genes of HNSC. (**A**) Uni-cox survival analysis list of immune-related features that significantly affect the prognosis of HNSC patients. (**B**) Forest plots of uni-cox survival analysis of Pseudotime and 5 module eigenvectors of HNSC. (**C**) Immune infiltration of class-switched memory B cells can significantly improve the prognostic survival of HNSC patients in Cluster1 and Cluser2 immune subtypes, but it is uncertain in Cluster3 immune subtypes. (**D**) Kaplan–Meier curves of differential brown module eigenvalues and prognoses in patients with HNSC; patients with high brown module eigenvalues had better overall survival (OS). (**E**) Kaplan–Meier curves of differential turquoise module eigenvalues and prognoses in patients with HNSC; patients with high turquoise module eigenvalues had better overall survival (OS). (**F**) Kaplan–Meier curves of differential class-switched memory B cell scores (xCell) and prognoses in patients with HNSC (logrank test). (**G**–**I**) Comparison of TIDE, dysfunction and exclusion scores for different immune subtypes. Comparison of scores of Cluster1, Cluster2 and Cluster3 immune subtypes. (**J**–**L**) Comparison of scores of Cluster1A, Cluster1B and Cluster2 immune subtypes. * *p* < 0.05, *** *p* < 0.001, **** *p* < 0.0001 and ns: not significant.

We conducted univariate Cox proportional regression analysis and identified Buffa Hypoxia Score and Winter Hypoxia Score as high-risk factors for the prognosis of HNSC patients (*p* < 0.001) [34]. B cells, CD4+ naïve T cells, CD8+ central memory T cells, CD8+ T cells, gamma delta T cells, memory B cells (*p* < 0.05), and immune infiltration of these immune cells were low risk factors for HNSC (Figure 9A). Of the six factors, Pseudotime, green, brown, turquoise, blue and yellow, Pseudotime was a high-risk factor for prognosis in HNSC patients (*p* < 0.01), while the brown and turquoise modules were low risk factors (*p* < 0.05) (Figure 9B). In addition, we examined the relationship between immune infiltration of class-switched memory B cells and the prognosis of HNSC patients in three immune subtypes. The results showed that immune infiltration of class-switched memory B cells was a beneficial factor in all three immune subtypes (HR = 0), although it was not significant in Cluster3 (*p* = 0.37) (Figure 9C). This may be related to the small number of samples in Cluster3. The heterogeneity of Cluster3 itself may also be the reason. Among HNSC patients, patients with higher brown and turquoise principal component vector values had better prognostic survival rates, which are significantly different from those in the low score group (*p* < 0.001) (Figure 9D,E). Similarly, patients with higher immune infiltration of class-switched memory B cells had better prognostic survival (*p* < 0.001) (Figure 9F). This suggests that immune infiltration of antibody-secreting B lymphocytes significantly improves the prognosis of survival in HNSC patients. Patients of all three immune subtypes benefited from immune infiltration of antibody-secreting B lymphocytes. Additionally, the immune infiltration of CD8+ T cell-related cytotoxic T cells can also significantly improve the prognostic survival of patients. For Cluster3 subtypes in a “cold” immune state, activating their humoral immune response through ICI therapy may have a positive effect on improving patient prognosis.

### 3.8. The Benefit of ICI (Anti-PD1 and Anti-CTLA4) Therapy in Different Immune Subtypes

**Figure 10:**
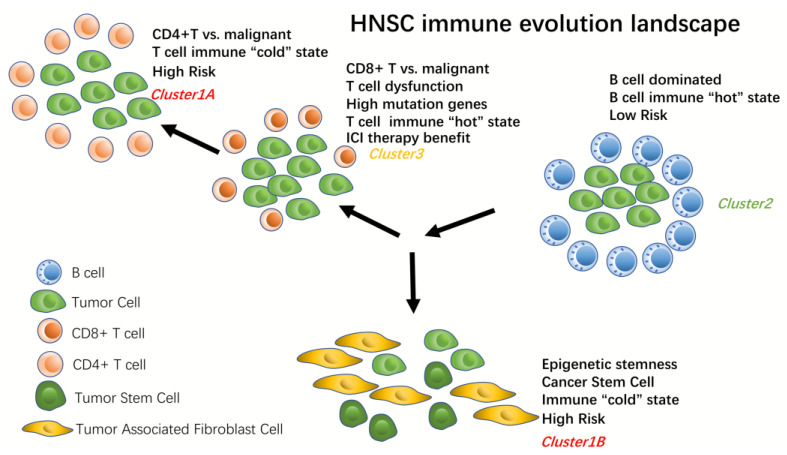
Molecular evolutionary landscape of the immune landscape of HNSC. Cluster2 is in the B cell dominant state, Cluster1A is in the CD4+ T dominant state, Cluster1B is in the fully deactivated T cell state (epigenetic stemness activation state) and Cluster3 is in the CD8+ T dominant state. However, Cluster2 has a higher survival rate, mainly because B lymphocytes are the dominant factor for prognostic survival in HNSC.

We assessed the therapeutic efficacy of immunotherapy in different HNSC immune subtypes using TIDE. Higher TIDE prediction scores imply a higher potential for immune escape, suggesting a lower benefit for patients to immune checkpoint (ICI) therapy. We evaluated the TIDE, T cell dysfunction and T cell exclusion scores of these immune subtypes in the immune subtypes obtained from consistent clustering and the immune subtypes obtained from the HNSC immune landscape, respectively (Figure 9G–I). The results revealed that Cluster3 had the highest TIDE and T cell dysfunction scores and the lowest T cell exclusion scores among the three subtypes, indicating the lowest benefit from immune checkpoint treatment. Conversely, Cluster1 had the opposite pattern. We observed that Cluster2 had a higher TIDE, T cell dysfunction score and lower T cell exclusion score compared to Cluster1A and Cluster1B (Figure 9J–L). This suggests that immune checkpoint therapy for Cluster2 is less effective. However, the prognosis of Cluster2 is the best of all immune subtypes. Furthermore, we found that Cluster1B had higher T cell exclusion than Cluster1A, which further demonstrates that tumor stemness increases and T lymphocytes also show inactivation as HNSC evolves to the Cluster1B state. Combining these analyses, we can outline the molecular evolutionary landscape of the tumor (Figure 10).

## 4. Discussion

Immunotherapy has emerged as a promising approach for the treatment of HNSC, but identifying the ideal immunotherapeutic subtype remains a challenge [35]. In this study, we classified HNSC into three intermediate immune subtypes based on the immune gene set of HNSC patients, thereby pinpointing the population for which the immunotherapy may be suitable. Each immune subtype exhibits unique immunological, cellular–molecular and clinical features. Cluster2, characterized by B-lymphocyte immune activation, has a significantly better prognosis compared to the other immune subtypes. Cluster1, with the highest hypoxia score, has the worst prognosis among the three subtypes. Cluster3 has the most mutated genes and is in an unstable state of immune microenvironment, between the “hot” immune state of Cluster2 and the “cold” immune state of Cluster1. The same conclusion was reached in the HNSC immune landscape. The high expression of ICP genes in Cluster2 and Cluster3 indicates that these two immune subtypes are in a state of immune activation, with Cluster2 in a state of B-lymphocyte immune activation and Cluster3 in a state of T-lymphocyte immune activation. The relatively high expression of ICD in Cluster1 suggests that the immune subtype of Cluster1 is more likely to be immunosuppressed and less responsive to immunotherapy. The ICD module genes were also expressed at high levels in Cluster3. This could explain the fact that Cluster3 is in an unstable state where both immune activation and immune suppression are at play. This state may allow for more mutations in the immune subtype of Cluster3, which may be associated with metastasis of HNSC cancers. The complex tumor immune microenvironment of HNSC indicates extensive heterogeneity between patients, which also helps us to design targeted immunotherapy regimens for individual immune microenvironment states. Therefore, we trained an SVM classifier based on 15 feature genes for classifying the immune subtypes of HNSC patients. The results show that this classifier has excellent performance in the validation set with a great generalization ability. In a later application, we can infer the immune subtypes of HNSC patients based on the expression profiles of the 15 genes and thus target immunotherapy strategies.

The immune microenvironment of the tumor determines the success of mRNA vaccines and immunotherapeutic strategies [36]. Therefore, we further delineate the immune subtypes of HNSC patients in more detail. We have mapped the molecular evolution of the immune subtypes of HNSC using monocle’s DDRTree algorithm. Within this map, we found that the distribution of the immune subtypes of HNSC gradually deteriorated from Cluster2 to Cluster1 in a continuous process. In Cluster1, two branches of HNSC immune subtypes emerged, Cluster1A and Cluster1B. Interestingly, Cluster3 is distributed during the divergence of Cluster2 to Cluster1A.This illustrates the distinct differentiation pathways between Cluster1A and Cluster1B. The evolution of Cluster2 to Cluster1B is accompanied by the fibrosis of the cancer cells and the “cooling” of the immune system. The molecular evolutionary landscape shows that the immune infiltration of secretory B lymphocytes (Cluster2) has a good prognostic value for HNSC patients, while the immune infiltration of T lymphocytes may lead to the differentiation of HNSC patients to the Cluster1A state through the evolution of Cluster3 immune cells and cancer cells against each other. Patients with a Cluster1B state of HNSC exhibit immune “cooling” accompanied by an increase in tumor epigenetic stemness, the most dangerous state for HNSC patients. The same conclusion can be drawn from the analysis of component1 and component2 and Pseudotime of the immune landscape diagram. By co-expression network analysis, we found five modules related to the immune system. We found that the brown and turquoise modules were most relevant to the molecular evolution of HNSC immune subtypes in the immune landscape. The co-expression network of the brown module was most activated in the Cluster2 immune subtype, whereas the co-expression network of the turquoise module was most activated in the Cluster3 immune subtype. By single cell transcriptome profiling, we localized the co-expression network of the brown module to plasma cells and the co-expression of the turquoise module to T cells.

Finally, we used the TIDE database to predict the responsiveness of these immune subtypes to immune checkpoint therapies. TIDE allows the identification of two mechanisms of tumor immune escape: the induction of T lymphocyte dysfunction in tumor settings with high infiltration of cytotoxic T lymphocytes (CTLs), and the blocking of T cell infiltration in tumors with low CTL infiltration. Numerous studies have shown that the TIDE score accurately predicts outcomes for patients with melanoma treated with anti-PD1 or anti-CTLA4. However, TIDE focuses on the function and status of T lymphocytes and does not fully reflect the complexity of the tumor immune microenvironment in response to immunotherapy. We found that the prognosis of HNSC was more relevant in relation to antibody-secreting B lymphocytes. Tumor immunity dominated by T lymphocytes does not appear to be as important in HNSC as humoral immunity mediated by B lymphocytes. This suggests that ICI-based immunotherapeutic regimens may have better efficacy in Cluster1. A B lymphocyte-mediated immunotherapy regimen could play a more active role in the treatment of HNSC.

## 5. Conclusions

In summary, by examining the immune subtypes of HNSC patients, we have charted the molecular immune evolution of HNSC patients, proposed a trajectory for the occurrence and development of immune subtypes in cancer patients, and suggested the possibility of molecular modulation of immune subtypes in HNSC patients with immune cells and immunotherapy. Our findings reveal a significant degree of heterogeneity within the cancer immune microenvironment of HNSC, indicating that immunotherapy could be employed as a means to intervene in the disease’s developmental process. Nevertheless, the targeted intervention on the immune evolutionary trajectory of the immune subtypes of cancer will need to be validated in future studies.

## Data Availability

Proteome profiling and clinical and transcriptome data about Head and neck cancer were downloaded from TCGA Database (https://portal.gdc.cancer.gov/) and GEO Database (https://www.ncbi.nlm.nih.gov/geo/). Please contact the author if you want to access the codes.

## Supplementary Materials

The following supporting information can be downloaded at: https://www.mdpi.com/article/10.3390/biom13071120/s1, Figure S1: Indicators of tumor stem cell assessment of HNSC molecular subtypes; Figure S2: Machine Learning Model Feature Extraction and Model Prediction; Figure S3: The Comparison of XCELL immuno-infiltration scores; Figure S4: The correlations between Module Membership and Gene significance in each module; Figure S5: GO function enrichment analysis; Table S1: 20 feature genes were filtered by SVMRFE algorithm to build classifiers for cluster1A, cluster1B, cluster2 and unclassified; Table S2: Three machine learning models (SVM, Random Forest and Naïve Bayes) were trained using 10-fold cross validation in the training set; Table S3: Performance of the model in the test set; File S1: Supporting information; File S2: Comparison of machine learning models in the validation set.

## Abbreviations

TCGA	The Cancer Genome Atlas
GEO	Gene Expression Omnibus
TIDE	Tumor Immune Dysfunction and Exclusion
GSEA	Gene Set Enrichment Analysis
HNSC	Head and neck squamous cell carcinoma
TISCH	Tumor Immune Single-cell Hub
WGCNA	Weighted Gene Co-expression Network Analysis
OS	Overall survival
TIME	Tumor immune microenvironment
ICPs	Immune checkpoints
ICD	Immunogenic cell death
DCs	Dendritic cells
APC	Antigen presenting cells
ICB	Immune checkpoint blockage
MDSCs	Myeloid-derived suppressor cells
