# Supplementary material for: Molecular Evolutionary Landscape of the Immune Microenvironment of Head and Neck Cancer
Source: Biomolecules. 2023 Jul 14;13(7):1120. doi: 10.3390/biom13071120 (PMC10377423; doi:10.3390/biom13071120)
Supplement: Supplementary file 1 [file biomolecules-13-01120-s001.zip › File S2.pdf]

10 feature genes were filtered by SVMRFE algorithm to build classifiers for cluster1, cluster2, cluster3

Genes	Info. gain	Gain ratio	Gini	ANOVA	χ^2	ReliefF	FCBF
**IGKV3-15**	0.718	0.359	0.341	281.704	200.320	0.149	0.752
**CD79A**	0.689	0.345	0.322	333.081	195.868	0.133	0.000
**IGKV1D-16**	0.631	0.316	0.310	276.325	185.964	0.118	0.000
**IGKV1-5**	0.618	0.309	0.304	289.303	182.599	0.146	0.000
**IGHV3-73**	0.566	0.283	0.279	261.686	172.754	0.101	0.512
**IGHV3-11**	0.632	0.316	0.308	291.050	185.769	0.133	0.607
**IGLV3-21**	0.609	0.304	0.286	275.190	180.843	0.129	0.000
**IGLV3-10**	0.523	0.262	0.251	212.245	161.904	0.140	0.455
**IRF7**	0.087	0.044	0.025	12.591	20.889	0.040	0.055
**TRBV7-4**	0.093	0.049	0.039	16.379	46.234	0.032	0.000

Three machine learning models (SVM, Random Forest and Naïve Bayes) were trained using 10 fold cross validation in the training set. (cluster)

Model	AUC	CA	F1	Precision	Recall
SVM	0.983	0.908	0.903	0.901	0.908
Random Forest	0.958	0.888	0.851	0.820	0.888
Naive Bayes	0.967	0.821	0.851	0.901	0.821

Performance of the model in the test set. (cluster)

Model	AUC	CA	F1	Precision	Recall
SVM	0.992	0.948	0.945	0.946	0.948
Random Forest	0.980	0.902	0.865	0.833	0.902
Naive Bayes	0.975	0.827	0.857	0.912	0.827
