# Supplementary material for: Molecular Evolutionary Landscape of the Immune Microenvironment of Head and Neck Cancer
Source: Biomolecules. 2023 Jul 14;13(7):1120. doi: 10.3390/biom13071120 (PMC10377423; doi:10.3390/biom13071120)
Supplement: Supplementary file 1 [file biomolecules-13-01120-s001.zip › Supporting information.pdf]

FigureS1

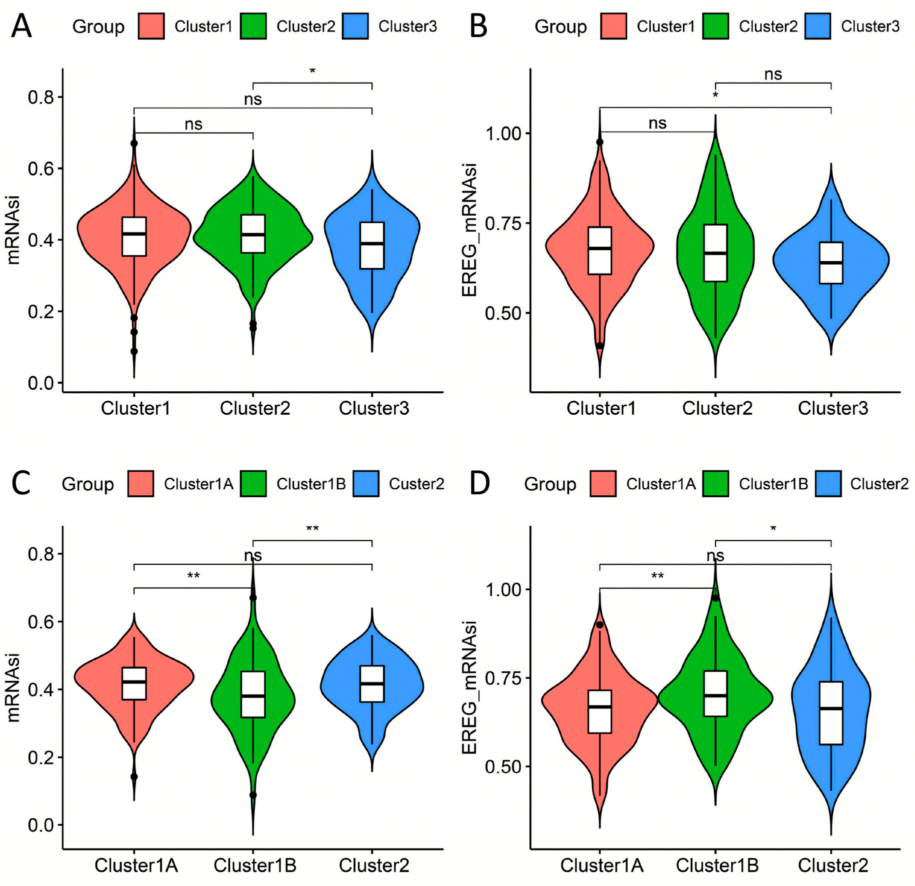

Figure S1. Indicators of tumor stem cell assessment of HNSC molecular subtypes. (A) Differences in mRNAsi between Cluster1, Cluster2, Cluster3. (B) Differences in EREG_mRNAsi between Cluster1, Cluster2, Cluster3. (C) Differences in mRNAsi between Cluster1A, Cluster1B, Cluster2. (D) Differences in EREG_mRNAsi between Cluster1A, Cluster1B, Cluster2. Wilcoxon Test, *P<0.05, **P<0.01, ***P<0.001, ****P<0.0001, ns: not significant.

FigureS2

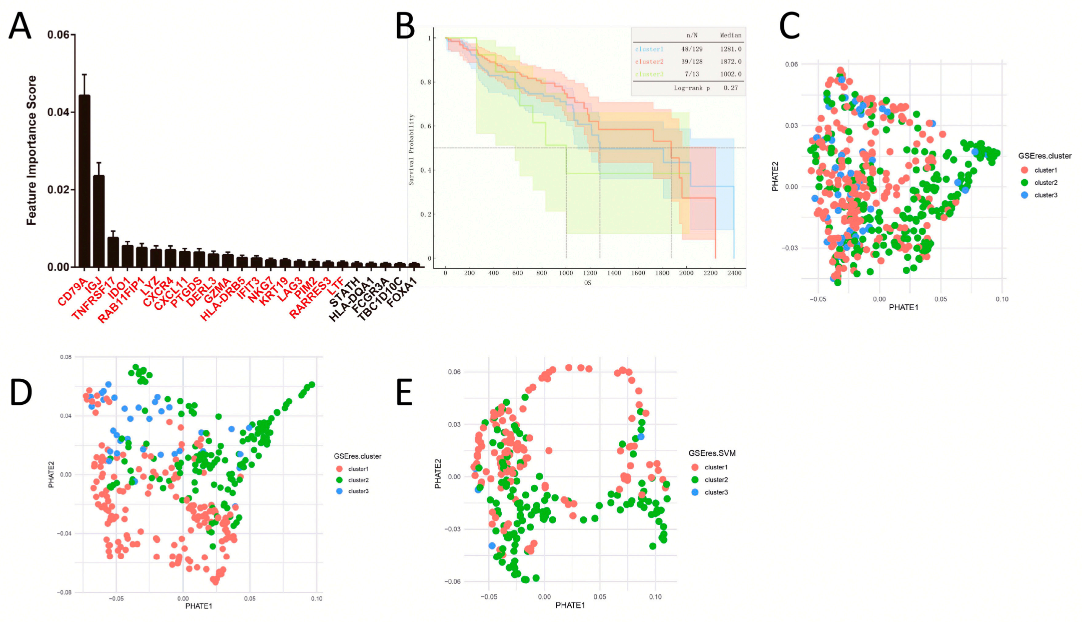

Figure S2. Machine Learning Model Feature Extraction and Model Prediction. (A) Feature selection. Weight statistics for top20 genes obtained using logistic regression (L2 regularization) permutation 1000 times in the training set of TCGA-HNSC. We screened the top15 genes as input features for the classifier (red markers). (B) Prognostic differences in immune subtypes of the GSE65858 cohort. (C) Phate two-dimensional scatter plot of 1090 genes with standard deviations greater than 1 for the TCGA-HNSC cohort. (D) Phate two-dimensional scatter plot of the 15 TCGA-HNSC signature genes. The graph shows that the three clusters can be clearly subgrouped. (E) Phate 2D scatter plot of 15 feature genes for GSE65858.

FigureS3

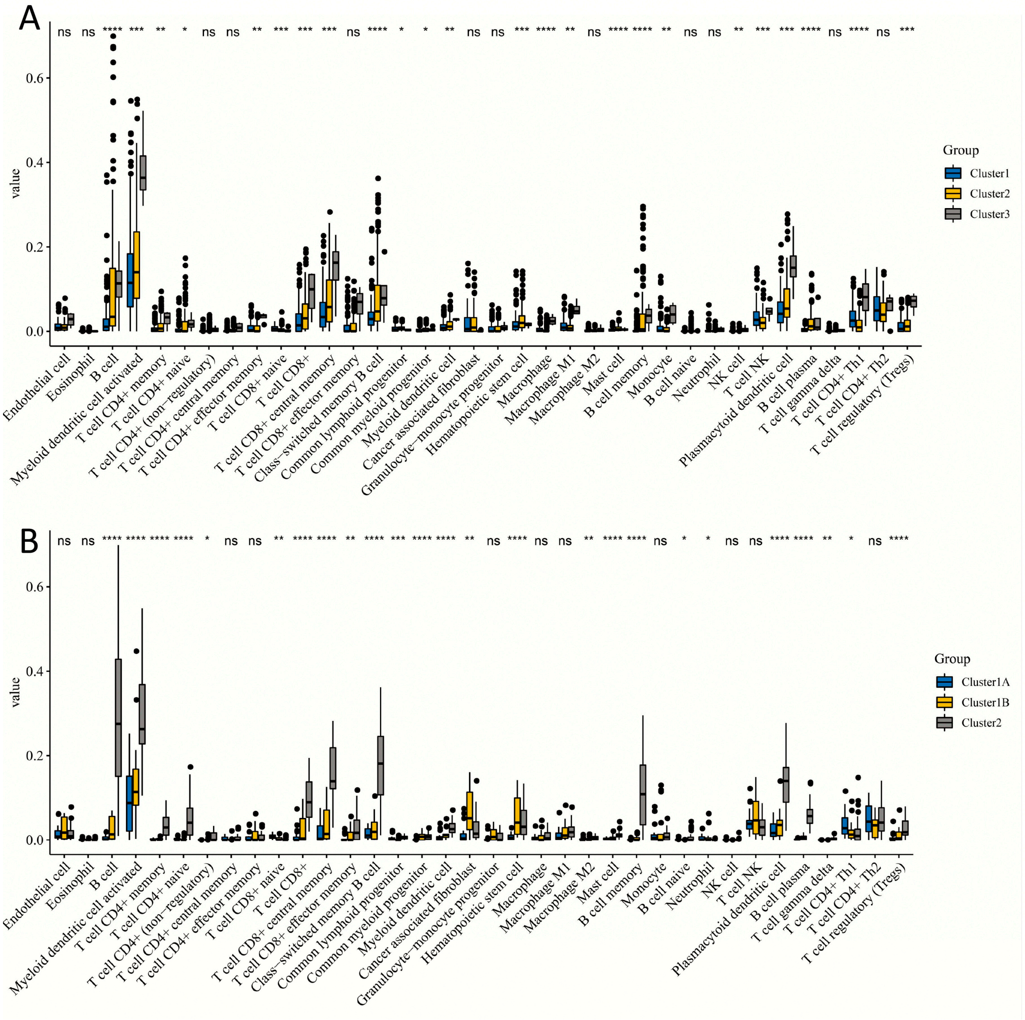

Figure S3. The Comparison of XCELL immuno-infiltration scores in Cluster1, Cluster2, Cluster3(A) and Cluster1A, Cluster1B, Cluster2(B) for the GSE65858 cohort (Kruskal-Wallis, *P<0.05, **P<0.01, ***P<0.001, ****P<0.0001, ns: not significant).

FigureS4

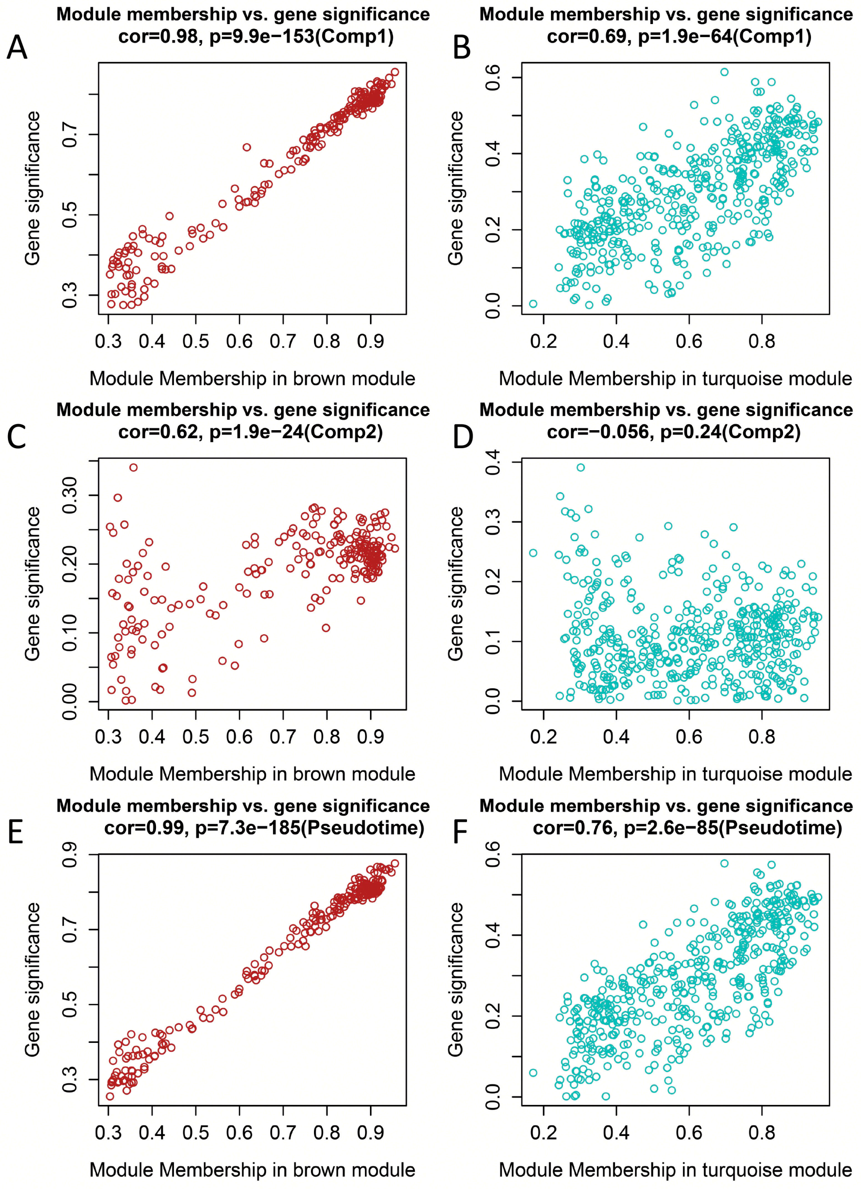

Figure S4. The correlations between Module Membership and Gene significance in each module. Gene significance indicates the person correlation coefficient of the genes in the module with the corresponding trait. Here we focus on the brown module and the turquoise module. (A, B) Comp1. (C, D) Comp2. (E, F) Pseudotime.

FigureS5

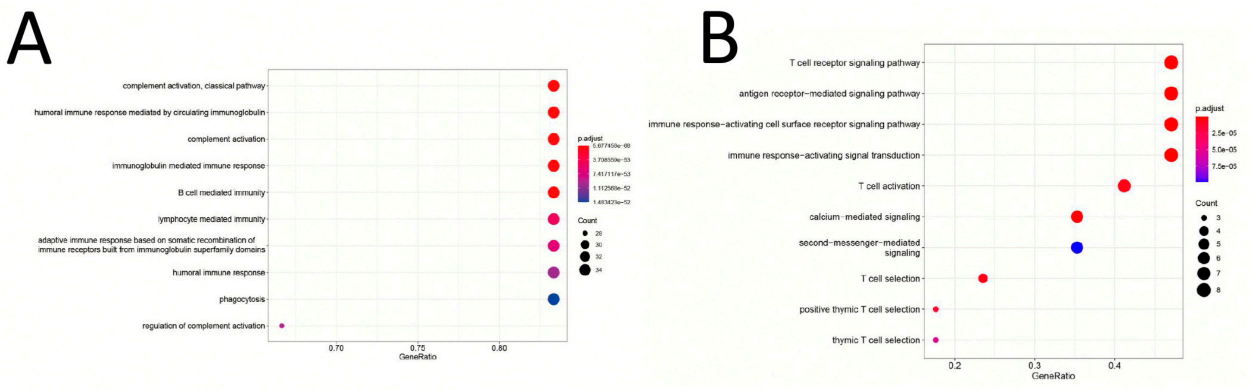

Figure S5. GO function enrichment analysis. (A) Brown module hub gene is mainly enriched in B-lymphocyte-dominated humoral immune-related signaling pathway. (B) The turquoise module hub genes are mainly enriched in T-lymphocyte-dominated cellular immune-related signaling pathways.

Table S1. 20 feature genes were filtered by SVMRFE algorithm to build classifiers for cluster1A, cluster1B, cluster2 and unclassified.

Genes	Info. gain	Gain ratio	Gini	ANOVA	χ^2	ReliefF	FCBF
**IGLV2-14**	0.688	0.344	0.329	309.079	196.499	0.104	0.700
**IGHV4-59**	0.678	0.339	0.317	346.723	193.454	0.168	0.000
**IGLV3-12**	0.568	0.284	0.273	154.444	174.996	0.061	0.000
**IGKV3D-11**	0.773	0.386	0.363	106.798	208.976	0.045	0.860
**IGKV6-21**	0.514	0.257	0.264	179.549	162.557	0.081	0.000
**IGLC3**	0.647	0.324	0.307	283.680	187.467	0.109	0.000
**IGLV1-40**	0.659	0.329	0.316	305.394	190.927	0.110	0.000
**IGHV3-48**	0.599	0.300	0.294	278.089	175.459	0.110	0.000
**IGLV4-3**	0.423	0.221	0.224	89.977	196.315	0.042	0.000
**IGLV8-61**	0.436	0.218	0.228	176.283	140.175	0.092	0.000
**NR3C2**	0.121	0.061	0.070	9.313	41.097	0.012	0.000
**CD22**	0.204	0.102	0.110	45.247	69.901	0.014	0.000
**TRAV35**	0.134	0.068	0.065	35.903	60.723	0.016	0.000
**TLR3**	0.072	0.036	0.018	18.843	23.101	0.031	0.045
**SLIT1**	0.038	0.019	0.023	0.930	13.403	0.002	0.000
**RBP4**	0.010	0.005	0.006	0.751	2.312	0.011	0.000
**TRAJ33**	0.029	0.045	0.012	5.669	20.779	0.002	0.000
**GIP**	0.027	0.016	0.008	3.118	9.754	0.000	0.018
**TRHR**	0.014	0.011	0.005	1.542	8.652	0.003	0.000
**TRAJ36**	0.034	0.074	0.014	5.258	21.488	0.005	0.000

Table S2. Three machine learning models (SVM, Random Forest and Naïve Bayes) were trained using 10-fold cross validation in the training set. (DDRT_cluster)

Model	AUC	CA	F1	Precision	Recall
SVM	0.943	0.784	0.781	0.779	0.784
Random Forest	0.928	0.741	0.689	0.646	0.741
Naive Bayes	0.930	0.746	0.748	0.751	0.746

Table S3. Performance of the model in the test set. (DDRT_cluster)

Model	AUC	CA	F1	Precision	Recall
SVM	0.979	0.890	0.889	0.891	0.890
Random Forest	0.960	0.772	0.719	0.675	0.772
Naive Bayes	0.948	0.772	0.772	0.772	0.772
